# Molecular Imaging in Primary Staging of Prostate Cancer Patients: Current Aspects and Future Trends

**DOI:** 10.3390/cancers13215360

**Published:** 2021-10-26

**Authors:** Reyhaneh Manafi-Farid, Shaghayegh Ranjbar, Zahra Jamshidi Araghi, Julia Pilz, Gregor Schweighofer-Zwink, Christian Pirich, Mohsen Beheshti

**Affiliations:** 1 Research Center for Nuclear Medicine, Tehran University of Medical Sciences, Tehran 1411713135, Iran; rmfarid@sina.tums.ac.ir; 2 Department of Nuclear Medicine, Division of Molecular Imaging and Theranostics, University Hospital Salzburg, Paracelsus Medical University, Muellner Hauptstrasse 48, 5020 Salzburg, Austria; s.ranjbar@salk.at (S.R.); z.jamshidi-araghi@salk.at (Z.J.A.); j.pilz@crcs.at (J.P.); g.schweighofer-zwink@salk.at (G.S.-Z.); c.pirich@salk.at (C.P.)

**Keywords:** prostate cancer, primary staging, PET/CT, [^11^C]choline, [^18^F]choline, [^11^C]acetate, [^68^Ga]Ga-PSMA, [^18^F]PSMA, [^111^In]In-capromab pendetide, [^99m^Tc]Tc-PSMA, [^18^F]Fluciclovine, [^11^C]Methionine, [^18^F]FDHT, gastrin-releasing peptide receptor, PET/MR, radiomics, sentinel lymph node biopsy

## Abstract

**Simple Summary:**

Accurate primary staging for determining the extent of prostate cancer is crucial for planning treatment in high-risk patients for distant metastases. Recurrence is still common after curative intent therapy, in spite of developments in the clinical nomograms for appropriate pre-treatment screening of patients for selective therapeutic approaches. This is partly due to suboptimal diagnostic performance of standard conventional imaging modalities such as computed tomography and bone scintigraphy. Molecular imaging by means of PET/CT and PET/MRI imaging using novel specific radiotracers might provide more precise staging of disease, allowing for better personalized treatments. This article reviews current developments and future trends for functional hybrid PET-targeted imaging in primary staging of prostate cancer.

**Abstract:**

Accurate primary staging is the cornerstone in all malignancies. Different morphological imaging modalities are employed in the evaluation of prostate cancer (PCa). Regardless of all developments in imaging, invasive histopathologic evaluation is still the standard method for the detection and staging of the primary PCa. Magnetic resonance imaging (MRI) and computed tomography (CT) play crucial roles; however, functional imaging provides additional valuable information, and it is gaining ever-growing acceptance in the management of PCa. Targeted imaging with different radiotracers has remarkably evolved in the past two decades. [^111^In]In-capromab pendetide scintigraphy was a new approach in the management of PCa. Afterwards, positron emission tomography (PET) tracers such as [^11^C/^18^F]choline and [^11^C]acetate were developed. Nevertheless, none found a role in the primary staging. By introduction of the highly sensitive small molecule prostate-specific membrane antigen (PSMA) PET/CT, as well as recent developments in MRI and hybrid PET/MRI systems, non-invasive staging of PCa is being contemplated. Several studies investigated the role of these sophisticated modalities in the primary staging of PCa, showing promising results. Here, we recapitulate the role of targeted functional imaging. We briefly mention the most popular radiotracers, their diagnostic accuracy in the primary staging of PCa, and impact on patient management.

## 1. Introduction

Prostate cancer (PCa) is the second most commonly diagnosed cancer and the fifth leading cause of cancer-related death in men [1]. The aggressiveness of PCa varies based on the primary risk stratification. PCa is traditionally stratified into low-, intermediate-, and high-risk, based on the sum of Gleason score, prostate-specific antigen (PSA) level, and clinical stage [2]. The five-year survival rate is nearly 100% for patients with localized PCa. However, it drops to 30% in those with distant metastasis [3]. Metastasis usually spreads through the lymphatics to the pelvic and para-aortic lymph nodes and hematogenous to the bones. Metastases to other organs (such as lungs and liver) are uncommon and related to the unusual pathology with a poor prognosis [4]. PCa is generally diagnosed using digital rectal examination, serum PSA level assessment, and transrectal ultrasonography (TRUS)-guided biopsy [2]. However, the diagnosis of clinically suspicious PCa is based on histopathologic confirmation. Histologically, the most common pathology in PCa is acinar adenocarcinoma [5].

Different imaging modalities are employed in the initial evaluation of PCa. Despite providing valuable information, there are some limitations. TRUS provides only a local field of view and is not sensitive enough for the detection of small primary lesions [2]. Computed tomography (CT) has limited value in the detection of lymph node (LN) metastasis and relatively poor performance in localizing early bone marrow metastasis [6]. Magnetic resonance imaging (MRI) is highly sensitive for primary tumor detection; however, again sensitivity is suboptimal for LN staging. Also, the limited field of view of MRI in the standard procedure overlooks metastasis outside the imaging area [6]. Functional imaging with bone scintigraphy has long been used for the assessment of bone metastasis with low specificity and poor sensitivity in low PSA levels [6].

Attempts were taken to find a PCa-specific agent, and a number of monoclonal antibodies were produced. Finally, [^111^In]In-capromab pendetide was employed by Wynant et al. in 1991 [7] and was approved soon after. However, the image quality and sensitivity were unsatisfying. Noteworthy, the prevailing oncology positron emission tomography (PET) tracer, 2-[^18^F]fluoro-2-deoxy-D-glucose ([^18^F]FDG) performed unsatisfactory in the vast majority of differentiated PCa [8]. Subsequently, new tumor imaging PET tracers, [^11^C/^18^F]choline and [^11^C]acetate, were developed, and their substantial uptake was depicted in PCa cells [9,10]. Hoping to overcome the limitations of previous modalities, several studies were conducted. However, moderate sensitivity and specificity were shown in the primary staging [11,12]. Later, small molecule prostate-specific antigen (PSMA) PET tracers were introduced and rapidly gained popularity [13]. PSMA is a metallopeptidase and a transmembrane glycoprotein overexpressed in PCa cell membranes, while PSA is a glycoprotein secreted into the lumen of prostatic ducts, which is over-released into the blood circulation following destruction of glandular architecture in PCa [14]. Meanwhile, ^99m^Tc-labeled tracers were also developed [15,16,17,18,19,20] to hypothetically provide PCa imaging cheaper and more extensively available.

Functional imaging (i.e., PET/CT and PET/MRI) using PCa-specific PET-tracers shows high accuracy in the biochemical recurrence status [21]. However, for the primary local staging, MRI and surgery are still the gold standards [2]. This might be related to the different tumor characteristics of the prostate cancer and mild to moderate uptake of PET-tracers (even specific ones) in the benign intra-glandular findings such as benign prostate hyperplasia and prostatitis, which may not be differentiable from cancerous tissues. Recent accessibility to hybrid PET/MRI systems is intriguing, providing simultaneous anatomical details and functional data, which may further increase diagnostic accuracy. Furthermore, the application of the ever-growing field of radiomics and machine-learning in PCa may lead to more accurate non-invasive primary staging, which may be comparable with histopathological findings.

Despite all developments in imaging, the non-invasive staging of PCa is still a challenge. Here, we recapitulate the role of functional imaging in the evaluation of primary PCa. We briefly mention the most popular radiotracers that have been implemented, and we review the diagnostic accuracy of the different methods in the primary staging, providing the results of meta-analyses, whenever available. 

## 2. Targeting Agents

Radioimmunoscintigraphic imaging with [^111^In]In-capromab pendetide was first employed by Wynant et al. in 1991 for the imaging of PCa [7]. The agent targets the intracellular domain of PSMA [22]. PSMA is a type II transmembrane glycoprotein, overexpressed on the surface of prostate cancer cells [23]. Scintigraphy with [^111^In]In-capromab pendetide had inherent drawbacks for imaging [24]; however, it outperformed CT and MRI in initial studies for LN staging [25] and was approved by FDA in 1996. The sensitivity and specificity of [^111^In]In-capromab pendetide for LN staging were reported 62% and 72%, respectively [25].

Soon after, a ^99m^Tc-labeled monoclonal antibody was also produced [26]. Subsequently, another anti-PSMA agent-J591 was developed, binding to the extracellular domain and producing less immune response [27]. It was labeled with ^111^In and ^89^Zr for imaging purposes [28,29]. Seeking better results, the mini-body IAB2M became available and labeled with ^89^Zr for PET imaging [30]. The development of PSMA ligand inhibitors, binding to a specific section on PSMA molecule, opened a new area for imaging and therapy of PCa and put monoclonal antibodies in a shadow. Various PSMA inhibitor ligands were synthesized and labeled with different radioisotopes [27]. These agents also became available for scintigraphy and single-photon emission computed tomography (SPECT) tracers [15,16,17,18,19,20].

Nevertheless, one of the urea-based agents, PSMA labeled with ^68^Ga and later with ^18^F, revealed astonishing initial results and dominated the clinical investigations. Hence, other agents are scarcely addressed in the literature. ^68^Ga-, ^18^F-, and ^99m^Tc-labeled PSMA will be discussed below.

### 2.1. [^18^F]FDG

For oncology PET imaging, [^1^^8^F]FDG is the most commonly used radiotracer. [^1^^8^F]FDG is an analog of glucose and is taken up by malignant cells based on their metabolic activity [31]. However, the low metabolism of the well-differentiated PCa, the overlap between [^1^^8^F]FDG uptake in malignant and benign lesions, as well as urinary excretion of [^1^^8^F]FDG, limit its application in PCa [32,33,34,35].

[^1^^8^F]FDG PET/CT may have some values in the setting of the advanced progressive disease, depicting some of the lesions with hypermetabolism [36,37]. Also, [^1^^8^F]FDG uptake may be seen in some aggressive primary tumors and LN metastasis [38]; nevertheless, it has a limited value for the diagnosis of the primary tumor and primary staging of LN or bone metastases [8,39,40]. The sensitivity of 37–52% has been reported for prostate lesions [38]. On the other hand, [^1^^8^F]FDG PET/CT harbors prognostic value. Higher intensity of tracer uptake has been correlated with higher glucose transporter-1 (GLUT1) expression [41], advanced stage [40], higher pathological grade [42], lower cancer-related survival [42], and disease progression [43].

Finally, any incidental [^1^^8^F]FDG uptake in the prostate gland should not be ignored. A meta-analysis by Bertagna et al. in 2014 showed that the prevalence of incidental uptake in the prostate gland is 1.8% [44]. Also, they demonstrated that the pooled risk of malignancy in those patients underwent further evaluation and biopsy is 17% and 62%, respectively [44].

In summary, [^1^^8^F]FDG PET/CT is of limited value in the evaluation of primary PCa. However, it provides prognostic information regarding tumor aggressiveness and patients’ outcome. In addition, incidental uptake in the prostate gland should be further evaluated to rule out probable PCa.

### 2.2. [^11^C/^18^F]Choline

Choline PET was first introduced in 1998 for the evaluation of PCa by Hara et al. [9]. The rationale to use radiolabeled choline in the evaluation of malignancies was the upregulation of the choline kinase in tumors leading to trapping choline in the cell membrane as phosphatidylcholine [45]. However, other mechanisms may be involved in PCa cells [45]. Two major radiotracers of choline have been commonly used in clinical practice, [^11^C]choline and [^18^F]choline [46].

[^11^C]choline showed superiority over [^18^F]FDG and gained popularity [47], and later, a multitude of studies assessed its diagnostic accuracy in the primary staging of PCa. In early studies, Farsad et al. demonstrated a sensitivity of 66% and specificity of 81% for the detection of the primary tumor [48]. Also, de Jong et al. reported the sensitivity of 80% and specificity of 96% for LN staging [49].

PCa cells also exhibited an increased uptake of ^18^F-fluorinated choline [50,51,52]. However, an early study depicted that [^18^F]choline is not helpful in localizing the primary tumor [53]. Later, several studies demonstrated that [^11^C/^18^F]choline is not a tumor-specific agent, and there is a significant overlap of the intensity of uptake between benign and malignant lesions [11]. In a meta-analysis by Evangelista et al., the pooled sensitivity and specificity of [^11^C/^18^F]choline PET/CT were 62.6% (95% CI: 54–70.6%) and 76.3% (95% CI: 65.4–85.1%), respectively, for the detection of the primary lesions [54]. Additionally, no remarkable association was noted between the [^11^C/^18^F]choline uptake of the primary tumor and histopathologic or laboratory parameters [55,56,57].

A number of further surveys evaluated its role in N- and M-staging. Beheshti et al. reported a patient-based sensitivity of 45% and specificity of 96% for the detection of LN metastasis [56]. The sensitivity was higher in larger LNs (66% for metastases larger than 5 mm) [56]. Subsequently, in a meta-analysis, Evangelista et al. demonstrated a pooled sensitivity of 49.2% (95% CI: 39.9–58.4%) and pooled specificity of 95% (95% CI: 92–97.1%) for the primary LN staging [58]. Later, compared to MRI, the node-based sensitivity of [^11^C/^18^F]choline proved to be superior to that of MRI, in both staging and re-staging status (51% [95% CI: 46–57%] vs. 39% [95% CI: 34–44%], respectively) [59].

Furthermore, in the evaluation of bone metastases in primary staging, [^18^F]choline detected marrow-based lesions without morphological abnormalities [60,61]. In a meta-analysis, Guo et al. showed a pooled sensitivity of 95% (95% CI: 85–100%) and specificity of 91% (95% CI: 83–100%) for the detection of bone metastasis using [^11^C/^18^F]choline [62]. In another meta-analysis including both primary staging and restaging, Zhou et al., depicted that the sensitivity of [^11^C/^18^F]choline PET/CT is comparable with MRI (87% [95% CI: 80–92%] vs. 91% [95% CI: 69–98%], respectively) although inferior to Na[^1 8^F]F PET/CT (96% [95% CI: 87–99]) [63].

Finally, hybrid [^18^F]choline PET/MRI imaging may provide additional information evaluating the different aspects of the malignant cells although no significant correlation has been found between functional parameters derived from MRI and [^18^F]choline PET [64,65]. [^18^F]choline multi-parametric (mp)MRI has increased the detection rate of primary PCa lesions in Gleason score ≥7, in some studies [66,67]. Also, [^18^F]choline PET/MRI may provide further information correlating with PSA level, Gleason score, etc. [67].

**Figure 1:**
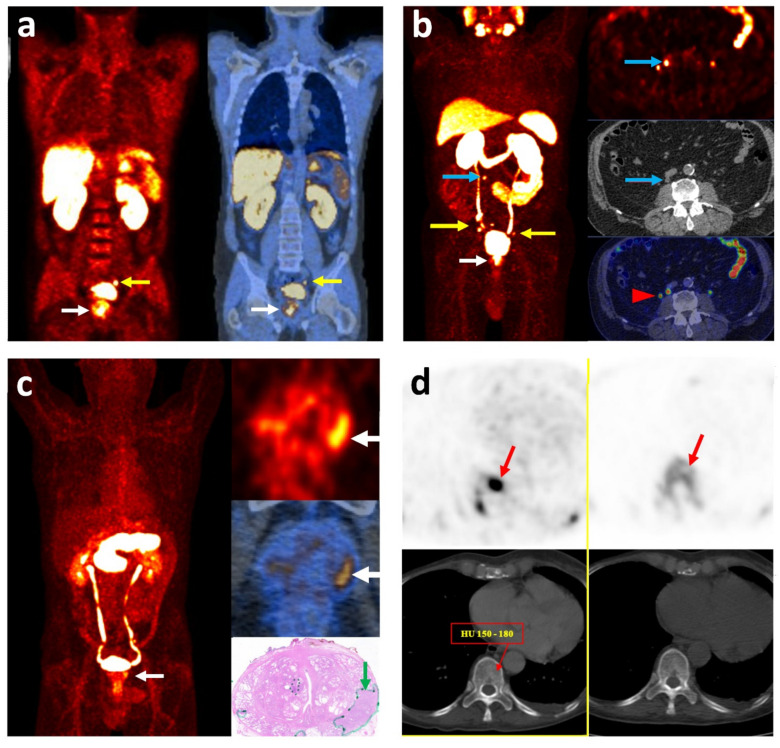
Imaging with different radiotracers localizing primary prostate cancer and its metastasis. (**a**) [^18^F]choline PET/CT: coronal fused images in a high-risk patient with GS = 8 and PSA = 9.6 ng/mL: primary lesion in the prostate gland (white arrow) and a metastatic lymph node in the left iliac chain (yellow arrow). (**b**) [^68^Ga]Ga-PSMA PET/CT: MIP (right) and transaxial (left) images in a high-risk patient with GS = 8 and PSA = 4.8 ng/mL: primary lesion in the prostate gland (white arrow) and multiple metastatic lymph nodes in the pelvis (yellow arrow). An unexpected small lymph node is also detected in the upper retroperitoneal region (blue arrow). The red arrowhead shows the physiologic activity in the right ureter. (**c**) [^68^Ga]Ga-RM2 PET/CT: MIP (right), transaxial (left) images and pathology section (right lower) in a high-risk patient with pT3a N1 (1/23) and GS = 9: primary lesion in the prostate gland (white arrow). The tumor is outlined with a green line and arrow in the pathology section. (**d**) Transaxial images of [^18^F]choline PET/CT (left) and [^18^F]Na PET/CT (right) in a high-risk patient, showing an early bone marrow metastasis (red arrow) without morphological changes on CT. GS: Gleason Score; MIP: Maximum Intensity Projection; PSA: Prostate-Specific Antigen.

Overall, keeping in mind that the role of [^11^C/^18^F]choline PET/CT is more prominent in the evaluation of biochemical recurrence [68], it had a suboptimal value in the initial staging of PCa. It showed limited sensitivity and specificity for the depiction of the primary tumor. Considering LN staging, it was not able to detect half of the malignant LNs, mainly the micrometastases (i.e., <5 mm), although the specificity was substantially high. The performance was acceptable for the detection of bone metastases but slightly inferior to the bone-specific agent, Na[^18^F]F PET/CT. However, it led to a change of the treatment approach in about 20% of high-risk PCa patients compared with the conventional imaging modalities (Figure 1a,b) [56]. Imaging with hybrid PET/MRI is a novel method warranting further studies to clarify its role.

### 2.3. [^11^C]Acetate

Acetate is a marker of metabolism. It is incorporated in the fatty acid synthesis associated with the cell membrane, reflecting high growth activity in malignant cells [69]. It has been more than two decades that [^11^C]acetate PET is introduced. Meanwhile, [^18^F] acetate was also developed, but due to initial unfavorable results, no further investigations proceeded [45].

In 2002, Oyama et al. reported successful imaging of primary PCa and its LN metastases with [^11^C]acetate PET [10]. In this primary study, they depicted a sensitivity of 83% for the advanced PCa [10]. From early studies, the non-specific accumulation of [^11^C]acetate was documented in the malignant and benign hyperplastic or normal prostate cells [70], which was confirmed in the following studies [71]. No correlation was demonstrated between biochemical (PSA) or histopathological (GS, FAS) findings or the intensity of uptake in the primary lesions [72,73,74]. Ultimately, in a meta-analysis, Beheshti et al. showed a pooled lesion-based sensitivity of 75.1% (95% CI: 69.8–79.8%) and specificity of 75.8% (95% CI: 72.4–78.9%) for the detection of primary tumor [12]. In the patient-based manner the pooled sensitivity was 93.0% (95% CI: 90.0–96.0%) [46].

The diagnostic accuracy for LN metastases is addressed less extensively in the literature [75]. Haseebuddin et al. demonstrated the sensitivity and specificity of 68.0% and 78.1%, respectively [75]. The respective values were reported 62% and 89% in LN-region-based manner by Schumacher et al. [76]. They also claimed that although the sensitivity was suboptimal for the detection of LN metastases, patients with positive PET had a higher rate of treatment failure [76]. Including the limited data, the pooled sensitivity was 73.0% (95% CI: 54.0–88.0%) in Beheshti et al.’s meta-analysis [12].

Considering distant metastases, skeletal in particular, the number of studies in the primary staging status is limited. It is mainly because patients with proven distant metastases in the initial staging were not included in those studies. In an early study, Omaya et al. showed that bone scintigraphy localizes 7 bone metastases, of which only 6 are detectable on [^11^C]acetate PET [10]. Contrarily, in a study by Strandberg et al., [^11^C]acetate PET/CT was superior to conventional bone scintigraphy in untreated high-risk PCa patients [77].

Investigating the additional value of hybrid PET/mpMRI, Polanec et al. showed that [^11^C]acetate PET/mpMRI improves the detection of primary lesions compared to MRI alone (sensitivity of 100% vs. 72.2%, respectively) [78].

In summary, similar to [^11^C/^18^F]choline, [^11^C]acetate has non-specific uptake in the prostate gland. It also misses a fraction of tumoral foci, limiting its value in the detection of primary PCa. Again, the accuracy for the detection of LN metastasis is suboptimal. Considering the accuracy in the detection of distant metastases, data is limited; however, it seems to have comparable or slightly higher sensitivity compared to bone scintigraphy in the detection of bone metastasis. [^11^C]acetate PET/CT is more applicable in the evaluation of biochemical recurrence with higher PSA values [79]. Finally, the addition of metabolic parameters of [^11^C]acetate PET to mpMRI in order to increase the accuracy of hybrid PET/MRI is an unsettled subject, requiring further investigations.

### 2.4. [^68^Ga]Ga-PSMA

Seeking after more favorable agents, a compound of small-molecule PSMA was developed (^68^Ga-labelled HBED-CC conjugate), which showed more affinity and specificity in binding to the PSMA-expressing PCa cells [80]. [^68^Ga]Ga-PSMA PET/CT was first employed in the clinic in 2012 [13]. Given its distinguished results, ^68^Ga-labeled PSMA rapidly gained popularity and was used in several surveys.

For the detection of the primary tumor, the performance of [^68^Ga]Ga-PSMA PET/CT showed promising results. Uprimny et al. reported patient-based sensitivity of 91% compared to the TRUS-guided biopsy [81]. However, it is known that TRUS-guided biopsy per se is suboptimal for the detection of primary PCa [82]. Several other publications addressed this issue, and ultimately in a meta-analysis, von Eyben et al. reported the pooled lesion-based sensitivity and specificity of 70% (95% CI: 53–83%) and 84% (95% CI: 24–99%) for the detection of primary tumor, respectively [83]. Expectedly, some small lesions are missed considering the limited spatial resolution of PET and the presence of background activity in the urinary tract. Also, the low sensitivity may be in part due to lower intensity of uptake in tumors with lower Gleason scores (i.e., <7) [84].

Some studies investigated the impact of the intensity of [^68^Ga]Ga-PSMA uptake in differentiation of the tumor. Correlations were shown between higher maximum standardized uptake value (SUV_max_) with Gleason score >7 and higher PSA level [81,85,86], as well as the presence of distant metastasis [87,88].

For T-staging and treatment planning of PCa, there is an established role for multiparametric (mp) and biparametric (bp) MRI [2]. In this regard, some studies evaluated the ability of [^68^Ga]Ga-PSMA PET/CT in determining the intra-prostatic location and disease extension. A meta-analysis by Woo et al. showed moderate sensitivity and high specificity of [^68^Ga]Ga-PSMA PET/CT for exhibiting local tumor extent [89]. Pooled sensitivity and specificity respectively were 68% (95% CI: 53–81%) and 94% (95% CI: 90–96%) for the seminal vesical invasion and 72% (95% CI: 56–84%) and 87% (95% CI: 72–94%) for the extra-prostatic extension [89].

Regarding N-staging, the reported range for sensitivity is variable [90,91]. Büdaus et al. were the first to evaluated the accuracy of [^68^Ga]Ga-PSMA PET/CT for the detection of metastatic LNs before radical prostatectomy [92]. While both patient- and node-based sensitivity were low (33.3% and 27.3%, respectively), the specificity was excellent (100%). The median size of detected LNs was 13.6 mm in their study [92]. Also, a recent study reported similar corresponding values of 30.6% for patient-based sensitivity and 95.6% for patient-based specificity with a median size of 7 mm for the detected LNs [93]. There could be an inevitable selection bias in the mentioned studies since they have included patients who are already candidates for radical prostatectomy with a lower risk disease. Furthermore, in a randomized trial (conventional vs. [^68^Ga]Ga-PSMA PET/CT), Hofman et al. showed that the sensitivity of [^68^Ga]Ga-PSMA PET/CT for N-staging is 85% [94].

For LN staging in intermediate- and high-risk patients, a recent meta-analysis found a high overall diagnostic value of [^68^Ga]Ga-PSMA PET/CT [95]. The pooled sensitivity was 84% (95% CI: 55–95%) and specificity was 95% (95% CI: 87–98%) [95]. It should be noted that the selection bias is also predictable in the studies included in this meta-analysis. Except for the selection bias, the variation in reported sensitivity in different studies could be due to the high dependence of the PET scan on the size of metastases in LNs.

From the view of M staging, studies have compared [^68^Ga]Ga-PSMA PET/CT with conventional methods. PCa has a propensity for bone metastases. Hence, most of the studies aimed to evaluate its role in the detection of bone metastasis. Compared with bone scintigraphy, which is considered the standard imaging modality in the setting of primary staging [2], [^68^Ga]Ga-PSMA PET/CT significantly outperformed bone scintigraphy (sensitivity was 97% vs. 86% and specificity was 100% vs. 87%, respectively) [96]. The other advantage of [^68^Ga]Ga-PSMA PET/CT over bone scintigraphy is the absence of flare phenomenon; however, it is not relevant in the setting of initial staging [97]. Also, the sensitivity of [^68^Ga]Ga-PSMA PET/CT was superior to MRI in terms of patient-based (97% vs. 91%, respectively) and lesion-based analyses (88% vs. 81%, respectively) [63]. However, [^68^Ga]Ga-PSMA PET/CT showed comparable diagnostic power with the high-sensitive Na[^18^F]F PET/CT [63]. The sensitivity and specificity for [^68^Ga]Ga-PSMA and Na[^18^F]F PET/CT were 97% (95% CI: 89–99%) vs. 96% (95% CI: 87–99%) and 100% (95% CI: 00–100%) vs. 97% (95% CI: 90–99%), respectively [63]. [^68^Ga]Ga-PSMA PET/CT also successfully detects visceral metastases [98,99,100].

Additionally, there are studies providing the extent of management alteration using [^68^Ga]Ga-PSMA PET/CT when it is performed for the primary staging, revealing promising results (Figure 1c). In different prospective and retrospective studies, it has changed management in 12.6–30% of patients [101,102,103]. In another prospective randomized trial, Hofman et al. found a 27% higher accuracy for initial staging and a higher rate of management change in 13% of patients using [^68^Ga]Ga-PSMA PET/CT compared to conventional imaging [94].

In summary, [^68^Ga]Ga-PSMA PET/CT detects primary PCa and provides accurate T-staging in approximately two-thirds of patients. Hence, it is not an ideal imaging method for the evaluation of primary prostatic lesions. In the detection of LN metastasis, although the performance of [^68^Ga]Ga-PSMA PET/CT is superior to other modalities, it cannot rule out the existence of N1 disease in approximately 15% of patients; therefore, extended pelvic lymph node dissection (ePLND) remains the gold standard for LN staging. For the detection of distant metastases, [^68^Ga]Ga-PSMA PET/CT is superior to conventional methods, mainly due to its accuracy for the detection of bone metastasis.

Although there are limitations for T- and N-staging, [^68^Ga]Ga-PSMA PET/CT impacts patient management. The results are substantial enough to be evaluated in future studies and to determine whether [^68^Ga]Ga-PSMA PET/CT is an essential modality for primary staging. Despite recent FDA approval, the application of [^68^Ga]Ga-PSMA PET/CT is still not explicitly mentioned in the guidelines [2,104]. Further studies evaluating the long-term impact of [^68^Ga]Ga-PSMA PET/CT or MRI seems necessary to clearly understand its role in the primary staging of PCa.

### 2.5. [^18^F]PSMA

As discussed before, PSMA ligands are playing an increasingly important role in the primary staging of PCa in intermediate- and high-risk patients [105]. The most widely used PSMA-based radiopharmaceutical is [^68^Ga]Ga-PSMA-11 [106]. [^68^Ga]Ga-PSMA-based PET was recently approved by FDA for PCa patients at initial staging and in the case of biochemical recurrence; however, it has some limitations [106]. In this regard, some new PSMA molecules labeled with other radioisotopes, such as ^18^F, were developed [107]. Fluoride-18 is a cyclotron-generating radioisotope that has more favorable characteristics than ^68^Ga, including production in larger quantities, transportation to satellite centers, longer physical half-life, delayed imaging, theoretically better spatial resolution [108,109,110], and possibly detection of smaller lesions due to lower positron energy [107,111]. Also, [^18^F]PSMA-1007 has biliary excretion. Non-urinary clearance may be advantageous for primary staging and in patients with possible local recurrence [112]. On the other hand, this may limit the ability of [^18^F]PSMA-1007 to detect liver lesions; however, the liver is not a common site for metastasis in PCa.

Except for [^18^F]PSMA-1007, there are other clinically available ^18^F-labeled PSMA agents ([^18^F]DCFPyL and [^18^F]DCFBC) [113,114,115,116]. Despite some differences, which may influence our preference for clinical use, all seem equally effective for imaging of PCa [111,112,117]. Very recently, [^18^F]FDCFPyL was also approved by FDA for staging and re-staging of PCa. Yet, there is no clear recommendation regarding which tracer should be selected [111].

From the initial studies, ^18^F-labeled PSMA PET/CT showed good accuracy in the detection of the primary lesions in the prostate gland [112,118,119]. Patient-based sensitivity ranged from 95 to 100% in different studies [120,121,122]. Moreover, associations between SUV_max_ and aggressiveness, higher GS or higher PSA level were demonstrated [120], but not in all studies [121].

In a head-to-head comparison of [^18^F]PSMA-1007 and [^68^Ga]Ga-PSMA-11 PET/CT, a perfect agreement was documented between two tracers in the detection of all dominant primary lesions [117,123]. However, the [^18^F]PSMA-1007 PET/CT could detect a few additional lesions [117,123].

For T-staging, [^18^F]PSMA-1007 PET/CT showed a good correlation with mpMRI and histopathology. It detected seminal vesicle invasion more than mpMRI (90% vs. 76%), while mpMRI was more accurate in detecting extracapsular extension (90% vs. 57%) [122], suggesting that hybrid [^18^F]PSMA-1007 PET/MRI would be a valuable modality for non-invasive T-staging.

In the early study of Giesel et al., the sensitivity and specificity (95% and 100%, respectively) were excellent for the metastatic LNs, including very small-sized ones (1 mm) [118]. In another, Sprute et al. compared [^18^F]PSMA-1007 PET/CT results with histology in 1746 LNs [124]. They reported node-based sensitivity of 71.2% and specificity of 99.5% [124]. Also, they found that the node-based sensitivity increases to 81.7% and patient-based sensitivity to 85.9% when only LNs larger than 3 mm are included [124]. Considering multidisciplinary consensus as the standard, Malaspina et al. showed significant superiority of [^18^F]PSMA-1007 PET/CT over conventional imaging with patient-based sensitivity of 87% and specificity of 98% [125]. [^18^F]FDCFPyL also showed the suboptimal sensitivity of 28.1–52.5% with excellent specificity of 94.0–99.4% for N-staging [126,127]. It seems that [^18^F]PSMA-1007 is more sensitive than [^18^F]FDCFPyL for N-staging. It may be due to the urinary elimination of [^18^F]FDCFPyL [128], which may obscure some small LNs in the pelvic cavity.

Regarding LN staging, ^18^F-labeled PSMA PET/CT shows variable sensitivity, depending on the node- or patient-based analyses. Also, it demonstrates excellent specificity. It may be in part due to the inherent higher resolution of ^18^F radioisotope and also low urinary excretion of ^18^F-labeled agents, theoretically allowing the detection of smaller LNs with a very low rate of false-positive findings.

Moreover, for the detection of bone metastases, Anttinen et al. compared [^18^F]F-PSMA-1007 PET/CT with conventional methods, reporting a clear superiority of [^18^F]PSMA-1007 PET/CT over bone scintigraphy, CT, SPECT/CT and whole-body MRI (area under curve (AUCs) were 0.90–0.91 vs. 0.71–0.8, 0.53–0.66, 0.77–0.75, and 0.85–0.67, respectively) [129]. They also revealed that [^18^F]PSMA-1007 PET/CT changes management in 18% of the patients [129]. Noteworthy, a substantial proportion of patients demonstrate non-specific bone lesions on [^18^F]PSMA-1007 PET/CT, which may be unrelated to PCa; hence, such lesions, especially when solitary, should be interpreted with caution to avoid overreading and improper treatment [130,131].

In summary, ^18^F-labeled PSMA agents are less comprehensively evaluated compared to ^68^Ga-labeled agents. ^18^F-labeled PSMA agents appear to be more promising, as they exhibit high labeling yield, excellent tumor uptake, and rapid, non-urinary excretion [118]. There is an increasing desire for ^18^F-labeled PSMA imaging in PCa, especially in centers with higher numbers of PCa patients [132]. However, there is no clear recommendation regarding which tracer should be selected [111].

^18^F-labeled PSMA PET imaging successfully detects primary lesions and metastases. Hypothetically better physical characteristics of [^18^F]PSMA-1007 PET/CT along with the non-urinary excretion could help detect smaller lesions in both prostate gland and metastatic LNs. The detection of smaller lesions has already been documented in a number of studies. For the primary lesions in the prostate gland, the detection rate is high. Perhaps dual imaging with mpMRI or with hybrid PET/MRI yields the best result. Also, for LN and distant metastases, ^18^F-labeled PSMA PET/CT outperforms conventional methods. The specificity is excellent for LN involvement. However, the sensitivity is reportedly variable for [^18^F]PSMA-1007 and [^18^F]FDCFPyL PET/CT. Head-to-head comparison will precisely determine the sensitivity of different tracers compared to each other. Future studies are mandatory to elucidate the accuracy of ^18^F-labeled PSMA PET imaging in the local staging, focusing on the important question, whether it can replace the standard ePLND. Finally, the non-specific uptake in bone lesions, with benign nature, must be considered with caution to avoid misinterpretation.

## 3. Other Agents

In addition to the discussed radiotracers, a number of other tracers have been used to explore other aspects of PCa, including amino acid transporters, androgen receptors, bombesin receptors, etc. These agents are briefly mentioned in this section.

### 3.1. [^99m^Tc]Tc-PSMA

The demand for PSMA-targeting PET imaging has increased significantly. However, PET/CT is a costly procedure and not widely available [133]. Therefore, different PSMA inhibitors were labeled with ^99m^Tc [134,135,136,137,138]. [^99m^Tc]Tc-PSMA scintigraphy has been investigated for different purposes, such as biochemical recurrence, primary staging, and radio-guided surgery [139,140,141,142,143].

One of the promising ligands is [^99m^Tc]Tc-MIP-1404, evaluated for primary staging using SPECT/CT in a few studies. The detection rate of 94–100% has been reported for the primary lesions [17,143,144], correlating with the Gleason score [17,143] and PSA level [17]. In a small report, the lesion-based sensitivity was 62.5% (25/40) for tumors with the Gleason score of 6 to 9 [145]. For LN staging, the sensitivity and specificity were 50% and 87%, respectively [143].

Limited studies have compared the diagnostic performance of [^99m^Tc]Tc-PSMA SPECT/CT with [^68^Ga]Ga-PSMA PET/CT [142,144,146,147], mostly including patients with biochemical recurrence [142,146,147]. Expectedly, all show superiority of PET/CT [142,144,146,147]. The detection rate was significantly lower in the prostate bed compared to extra-prostatic regions, in studies evaluating biochemical recurrence [142,147], while the primary lesions were localized with high accuracy [144]. Lesions in biochemical recurrence are smaller, so theoretically harder to be visualized in SPECT/CT images with inherent limited spatial resolution.

Overall, [^99m^Tc]Tc-PSMA SPECT/CT is not an ideal modality for primary staging. Due to the encouraging detection rate of the primary lesions, it might be used for guided biopsy for patients with high suspicion and negative biopsies, in patients with undetermined imaging findings or in regions with unavailable mpMRI or PET/CT facilities. In the biochemical recurrence, it might be helpful in radio-guided surgery or the evaluation of extra-prostatic regions. The most logical application of [^99m^Tc]Tc-PSMA SPECT/CT seems to be documentation of PSMA avidity before radioligand therapy.

### 3.2. [^11^C]Methionine

Similar to other malignancies, the increased activity of amino acid transporters was considered as a target to depict PCa lesions. [^11^C]Methionine shows minimal urinary excretion with low background activity in the pelvic cavity [148]. [^11^C]Methionine PET/CT has been used to detect primary lesions [148,149] and differentiate significant lesions (Gleason score >5) from non-significant ones (Gleason score ≤5) [150]; however, the results were unsatisfactory.

### 3.3. [^18^F]Fluciclovine (FACBC)

[^18^F]Fluciclovine, also known as FACBC (trans-1-amino-3-18F-fluorocyclobutanecarboxylic-acid), is a synthetically labeled amino acid [151,152]. The ability of [^18^F]Fluciclovine PET/CT to characterize primary tumors is somehow limited due to the overlap between the intensity of tracer uptake in the benign and malignant lesions [151,152]. The pooled specificity was less than 50% for the prostate bed lesions (combined primary and recurrent lesions) [153]. Additionally, it has shown comparable sensitivity with conventional imaging in the detection of LN [154,155,156] and bone [157] metastases. Similar to other PET tracers, the specificity was high for metastatic LNs [154,155].

[^18^F]Fluciclovine PET/CT performed better in the evaluation of recurrent disease [158] and was approved by FDA in 2016 [153]. However, later, it was shown to be inferior to [^68^Ga]Ga-PSMA PET/CT [159]. Hence, it seems that [^18^F]Fluciclovine PET/CT has no significant role in the primary staging of PCa, and will be replaced by new PSMA targeting agents for biochemical recurrence in guidelines.

### 3.4. Androgen Receptor

Additionally, androgen receptor expression has been assessed using 16beta-18F-fluoro-5-alpha-dihydrotestosterone [^18^F]FDHT PET/CT [160]. In a study by Dehdashti et al., the sensitivity and lesion detection rates of [^18^F]FDHT PET/CT were reported to be 63% and 86%, respectively [161]. The positive scan results correlated with higher PSA levels [161]. Additionally, the sensitivity was reported to be inferior to that of [^1^^8^F]FDG PET/CT [162]. As discussed in other studies [163,164], it seems that [^18^F]FDHT PET/CT may have a more significant role in the management and prognostication of advanced PCa rather than initial staging.

### 3.5. Gastrin-Releasing Peptide Receptor

Another imaging probe targets gastrin-releasing peptide receptor (GRPR), from the bombesin receptor family [165,166]. It is overexpressed in most PCa cells [167]. Bombesin increases the potential of invasion and migration of PCa [168,169]. Various GRPR agonists and antagonists have been synthesized and labeled with different radioisotopes, among which antagonists show superior imaging characteristics [170]. RM2 labeled with ^68^Ga is one of the antagonists with promising results, introduced in 2011 [171] and clinically used in 2013 (Figure 1d) [172]. Kähkönen et al. showed a sensitivity of 88% and specificity of 81% for the evaluation of primary lesions and sensitivity of 70% for LN metastasis [173]. Also, Beheshti et al. showed an encouraging lesion-based sensitivity of 81% with [^68^Ga]Ga-RM2 PET/CT for the primary lesions in the prostate gland, which was higher compared to [^18^F]Choline PET/CT (68%) [174]. Other promising antagonists, [^68^Ga]Ga-RM26 [165] and [^68^Ga]Ga-SB3 PET/CT [175,176], have also been shown to be safe and effective in the detection of primary and metastatic PCa.

Given promising results of targeting GRPR, a plethora of newer agents are being developed [175,176,177,178] to find an optimum radiotracer. Additionally, due to the heterogeneous expression of PSMA and GRPR in PCa cells, the concept of heterodimeric targeting of PCa is being investigated [179,180]. GRPR antagonists have drawn the attention of multiple investigators. Further studies will shed light on their precise role in PCa, especially opposing or along with PSMA-targeting agents. Summary of diagnostic performance, advantages, and disadvantages of different radiotracers in the evaluation of the primary staging of PCa are provided in Table 1.

### 3.6. Urokinase Plasminogen Activator Ligand

Another promising agent is the urokinase plasminogen activator (uPA) ligand as a marker for aggressiveness [182,183]. Its uptake has been documented in primary lesions of PCa [184]. PET/MRI targeting uPA receptors has shown a correlation with Gleason score and may play a role in the non-invasive evaluation of primary prostate lesions [185].

### 3.7. VAPAC1-Targeting Agent

Overexpression of VAPAC1 receptors is seen in malignant lesions, including PCa [186], which can be used as a target for imaging of tumoral lesions. VAPAC1 PET/CT has been shown encouraging results in this regard in PCa [187], warranting further investigations.

### 3.8. α_v_β_3_ Integrin-Targeting Agent

The α_v_β₃ integrin plays a role in the invasion, metastasis formation and angiogenesis [188]. It is overexpressed in PCa [188]. However, the preclinical studies did not show remarkably distinguished uptake using ^18^F/^68^Ga-labaled RGD PET/CT [189,190].

## 4. PET/MR

The high sensitivity of MRI for the evaluation of primary PCa is well-known [2]. Therefore, the hybrid PET/MRI system became an appealing modality in PCa since its first introduction in 2010 [191]. Soon after, the mpMRI, also evaluating the functional characteristics, became forward as a powerful modality in localizing tumors. Currently, its efficacy is increasingly investigated in PCa.

The performance of PET/CT and PET/MRI was comparable in some studies [192,193]; however, in others, PET/MRI outperformed PET/CT or MRI images alone [67,194,195,196]. For example, Eiber et al. compared PET/MRI with mpMRI for PCa localization [194]. They showed that [^68^Ga]Ga-PSMA PET/MRI is superior to mpMRI for both detection and localization of the primary lesions [194]. Li et al. compared PET/MRI (including mostly [^68^Ga]Ga-PSMA) with mpMRI in a meta-analysis and reported that lesion- or region-based accuracy of PET/MRI is higher than that of mpMRI with AUC of 0.93 (95% CI: 0.89–0.96) vs. 0.84 (95% CI: 0.78–0.89), respectively [197]. Likewise, in two other meta-analyses evaluating only [^68^Ga]Ga-PSMA in one and different tracers in the other, PET/MRI showed high patient-based sensitivity [198,199].

For the extent of the local tumor PET/MRI seems to be a sensitive method, especially the MRI component [200]. The reported sensitives range from 66% to 94% for the extracapsular extension or seminal vesicle invasion [201,202]. Also, Muehlematter et al. demonstrated a slightly better accuracy for [^68^Ga]Ga-PSMA PET/MRI compared to mpMRI for the detection of the extracapsular extension [203]. The accuracy was similar for seminal vesicle infiltration [203]. The specificity of PET/MRI was slightly lower than mpMRI for both [203].

Furthermore, for the evaluation of LNs, a few studies reported patient-based values. The range for sensitivity was from 60 to 68.8% and for specificity was from 95 to 100% [201,202,204,205]. Noteworthy, all missed LNs (5/16) were smaller than 4 mm in the study by Grubmüller et al. [202], and the mean size of the missed LNs was 2.7 mm in van Leeuwen et al.’s report [205].

For the evaluation of distant metastasis, whole-body PET/MRI and PET/CT apparently have similar performances. Studies show a high correlation between two scans for lesion detection [192,206,207]. However, anatomical delineation might be better on PET/MRI [192].

Overall, standalone PET/MRI appears to be of great value for preoperative staging of PCa. Due to high patient-based sensitivity and specificity, PET/mpMRI could have a substantial role in guided biopsy of the prostate gland. Also, PET/MRI seems to be highly sensitive for T-staging. However, the specificity seems suboptimal [203]. Additionally, despite excellent specificity, PET/MRI still misses small metastatic LNs. The sensitivity is moderate for N-staging; hence, it presently cannot preclude the invasive surgical N-staging. For the evaluation of distant metastases, the limited available studies show comparable performance for both PET/MRI and PET/CT. However, there are a number of technical merits and demerits for performing whole-body PET/MRI [208]. Currently, we believe that hybrid PET/MRI would be of more value for the evaluation of the primary lesions in the prostate gland. Further studies are required to determine the cost-effectiveness of whole-body PET/MRI in the setting of primary staging.

## 5. Radiomics

The current high-quality modalities provide valuable images for qualitative assessment of the tumoral lesions. Quantitative and semiquantitative analysis can reveal further characteristics of lesions, which are not assessable with human eyes. Beyond these fundamental data, there is extensive information, so-called features, embedded in the images. Radiomics is a method used in medicine to extract the features from medical images and unravel additional hidden characteristics. These features correlate with relevant genetic, pathologic, clinical, or prognostic features [209]. Given the enormous number of features, machine-learning algorithms are also employed for data analysis [210].

The detection of the primary PCa is still a challenge. Mp-MRI has a high sensitivity (93%, 95% CI: 88–96%) for the detection of PCa; however, the specificity (41%, 36–46%) is poor, and there is a 10% false-negative result [211]. There is hypothetically spatial heterogeneity in the malignant lesions [212], which cannot be visualized or detected with usual quantitative parameters. Hence, radiomics has been engaged in the field of PCa.

A few studies evaluated the radiomics features of the primary lesion to discriminate the primary tumor and predict adverse features. Machine-learning models have been shown to correlate with human readers in detecting primary PCa [213,214]. They also have improved diagnostic accuracy [215]. Also, the analysis of radiomics features could discriminate lesions with Gleason score 7 and ≥8, as well as predict LN positivity with AUC of >0.84 [213]. Moreover, machine-learning models outperformed standard PET parameters and predicted LN status (AUC 0.86), metastasis (AUC 0.86), Gleason score (AUC 0.81), and extracapsular extension (AUC 0.76) [216]. They could discriminate active and responded sclerotic bone metastases on CT (AUC 0.76) [217]. Also, they were significantly superior over known clinical, laboratory and histopathological adverse features in predicting biochemical recurrence (AUC 0.90) and overall patient risk (AUC 0.94) [218]. Additionally, a recent study reported predictive values of the radiomics (derived from metabolic tumor and peripheral zones) for Gleason score, PSA group, TNM stage, and progression-free survival [219].

Overall, radiomics and the application of machine-learning in medicine are rather novel practices. Radiomics is increasingly employed in the field of radiology and nuclear medicine. Hypothetically, it will have a compelling impact on individualized medicine. However, there are numerous technical challenges [220]. Considering PCa, radiomics has shown promising results in delineating primary tumors and predicting stage and outcome. Further studies are required to assess the different technical aspects, approaches, and the definite clinical role of radiomics in PCa.

## 6. Sentinel Lymph Node Biopsy

Sentinel lymph node biopsy (SLNB) theoretically detects the first LNs in the chain of lymphatic drainage of the primary tumor [221]. The expected benefits of SLNB are the reduction in the surgical time, cost, and potential complications, as well as an improvement of the staging by identifying unusual drainage roots [221,222]. SLNB was used in PCa in 1999 [223] and proved to be a valid method for N-staging with high diagnostic accuracy [224,225,226].

In a large study of 2020 patients with localized PCa, Holl et al. demonstrated a detection rate of 98.2% for SLNs [226]. The false-negative result is an important issue. In this regard, in a systematic review, Wit et al. calculated the overall false-negative rate to be 4.8% (0–18.2%) [225]. Additionally, there is evidence that the hybrid tracer technique using indocyanine green-^99m^Tc-nanocolloid improves the detection of SLNs [227,228,229,230]. On the other hand, studies showed that ePLND does not necessarily provide complete resection of involved LNs [230,231]. Metastatic LNs were missed in approximately 8–10% of patients undergoing ePLND without SNB, mainly due to metastases outside the template surgery [230,231]. Importantly, large non-randomized studies indicated that the biochemical-free [230,232] and clinical-free [230] survival rates are higher in patients undergoing SLNB.

The preferred tracer is ^99m^Tc-nanocolloid [226,229]. Performing SPECT/CT provided useful anatomical information and might result in more SLN detection, especially near the prostate gland and beyond the area of ePLND [233]. Also, lymphoscintigraphy with a PET-tracer, ^68^Ga-nanocolloid, successfully depicted SLNs and outlined aberrant drainage into the pelvic bones and perivesicular, mesorectal, inguinal, and Virchow nodes [234].

Overall, SLNB seems to provide clinical benefit in the primary staging of PCa; however, it demands additional equipment, expenditure and expertise. Further randomized controlled trials are necessary to clarify its additional clinical value compared to the standard procedures.

## 7. Discussion

**Figure 2:**
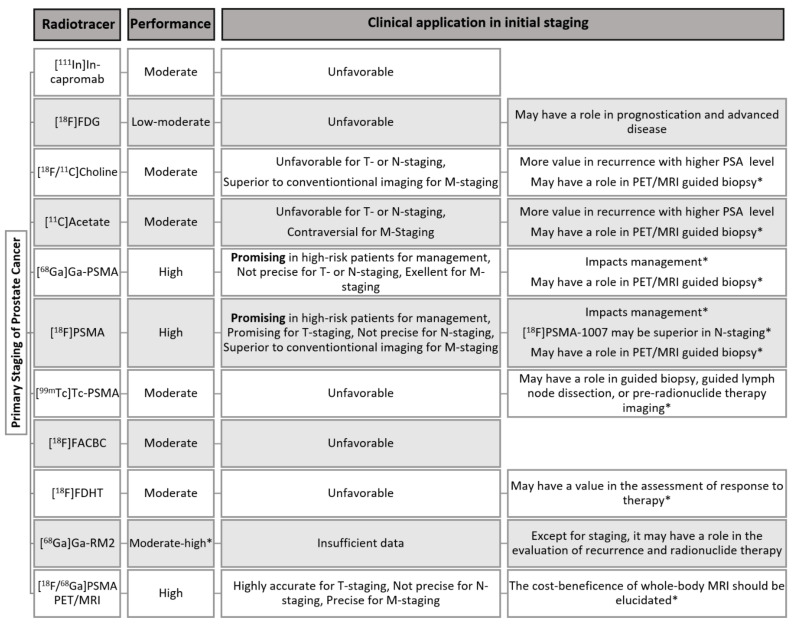
A summary of the role of nuclear medicine in the initial staging of prostate cancer. * The statements are based on limited but promising initial data, warranting further investigations.

The ultimate purpose of imaging is the accurate detection and staging for proper therapy. Nuclear medicine has an indisputable role in the management of PCa. Numerous tracers have been investigated for the evaluation of primary PCa, among which PSMA-targeting PET-tracers has shown more accuracy in every aspect of PCa imaging [21,63,159,235,236,237]. A summary of the role of different tracers in the evaluation of the primary staging of prostate cancer is provided in Figure 2.

First, the idea of early non-invasive detection of primary lesions is always compelling. Despite the impressive evolvement in PCa targeted radiotracers, from the early monoclonal antibodies to [^68^Ga]Ga-PSMA PET/CT, the accuracy never reached an ideal level to preclude invasive methods [83]. A part of this is due to the limited spatial resolution of the imaging equipment and high background activity in the pelvic region. Also, the inherent heterogeneity of the malignant cells in aggressiveness and receptor expression [179,180] further complicates the detection of small lesions. On the other hand, the ^18^F-labled PSMA PET imaging shows very promising results in the primary tumor detection and T-staging. It seems advantageous in the pelvic cavity owing to non-urinary excretion. Also, it detects more lesions compared to ^68^Ga-labeled counterpart. Further studies will determine whether ^18^F-labled PSMA tracers would prevail in the PCa imaging. Of note, the rate of ^18^F-labled PSMA uptake in benign lesions has been reported to be high in the re-staging status [238], which may require more knowledge and experience for precise interpretation.

Recent developments in mpMRI and engaging it with the functional data of PET have shown truly promising results. MpMRI is known for its high spatial resolution and detection rate [2,211]. Interestingly, some studies have shown higher accuracy for hybrid PET/MRI compared to mpMRI [67,194,195,196]. Additionally, the novel field of radiomics has recently been exploited in PCa [215] and is expected to increase the accuracy of interpretation and prediction of the stage and outcome. Noteworthy, invasive biopsy has a significant false-negative rate [82], which can be reduced by the guidance of imaging. The role of PET/CT, PET/MRI and radiomics needs to be clarified for the guided biopsy of the prostate gland. Also, more studies are necessary to evaluate the optimum diagnostic power of hybrid PET/MRI using the most accurate tracers and enrolling radiomics for primary tumor detection.

Second, after the detection of a malignancy in the prostate gland, precise T-staging is crucial for an optimal management and prognostication. Rather similar to the primary tumor detection, PET-only has limited spatial resolution compared to MRI for T-staging. Again, the application of hybrid PET/MRI [201,202,203] and radiomics [216] may increase the certainty.

Third, the LN staging is another debatable concern in PCa. The ePLND is recommended in intermediate- and high-risk PCa patients with a risk of LN invasion > 5% cutoff of the Briganti nomogram (or >7% in the updated nomogram), although this threshold misses 1.5% of patients with LN metastasis [2,231,239]. In addition, a significant number of patients may unnecessarily undergo invasive staging, which is associated with the risk of complications [225,240]. In a further aspect, due to the wide variation in lymphatic outflow, nodal metastases may appear beyond the standard lymphadenectomy templates (8–10% of cases) [230,231], which potentially leads to under-diagnosis and under-treatment. The therapeutic benefit of ePLND is also still debatable [241]. A recent systematic review concluded that ePLND does not improve oncologic outcomes [242]. These limitations emphasize the need for sensitive imaging techniques with limited false-negative results to improve the detection of LN metastasis and eliminate the need for unnecessary invasive procedures [243,244].

Currently, conventional imaging cannot compete with PSMA-targeting PET-tracers in the evaluation of LN metastasis; however, PSMA PET/CT still overlooks N1 disease in approximately 15% of the patients [94,95,124]. The node-based sensitivity is even lower. The size of the metastasis is a major influential factor. It is still unclear to how extent these small missed LNs would impact outcome since usually all patients undergo ePLND, which also practically fails to remove all involved LNs. Merging the high-quality data of mpMRI with PET and exploiting radiomics is expected to further increase the predictive potential of PET/MRI for LN metastasis. It would be of value to compare the predictive ability of radiomics with established nomograms for LN involvement, which may more individualize the surgical approach.

For precise LN staging, SLNB has also been successfully used in PCa, but it has not gained enough popularity. Although it demands additional equipment, expenditure and expertise, SLNB provides a higher detection rate [230,231]. Further randomized controlled trials are necessary to clarify its additional clinical value compared to the standard procedures.

Fourth, standalone PET/MRI appears to be of great value for preoperative staging of PCa. For the evaluation of distant metastases, the limited available studies show comparable performance for both PET/MRI and PET/CT [192,206,207]. Considering high cost and limited availability, the cost-effectiveness of whole-body PET/MRI in the setting of primary staging is yet to be established in future studies.

Fifth, the ultimate purpose of imaging is accurate management. The value of PSMA PET/CT is more established in the detection of recurrent disease [245,246] rather than primary staging. Noteworthy, PSMA-targeting imaging can change management in 12.6–30% of patients [101,102,103,129]. However, it is not yet a standard procedure in the setting of primary staging.

Despite recent FDA approval, the application of PSMA PET/CT in primary staging of PCa is still not explicitly mentioned in the guidelines [2,104]. Further studies evaluating the long-term impact of PSMA-targeting PET imaging is necessary to determine its role in the primary staging of PCa.

Sixth, other tracers are continuously introduced to evaluate the different aspects of the PCa and may find a role in PCa management. [^11^C]Methionine, [^18^F]Fluciclovine (FACBC), and [^18^F]FDHT have shown limited application in the primary staging. Also, [^99m^Tc]Tc-PSMA SPECT/CT demonstrated a circumscribed application in primary staging and may be helpful in other indications in areas with unavailable PET. However, early studies using various GRPR-targeted tracers have demonstrated promising results. Also, PET/CT targeting uPA and VAPAC1 is encouraging, requiring further studies.

## 8. Conclusions

Molecular imaging using nuclear medicine modalities plays a crucial role in the management of PCa. Various tracers have been employed, among which PSMA-targeting PET-tracers outperformed the others. Despite the high accuracy, the role in primary lesion detection and T-staging is still limited. Nevertheless, the ^18^F-labeled tracers show higher accuracy and may overcome this limitation. Also, the hybrid PET/MRI systems show superior diagnostic accuracy for the evaluation of prostate bed, even when compared to mpMRI. Additionally, the concept of PET/MRI-guided biopsy has become forward as an interesting field for future investigations. For N-staging, the sensitivity is acceptable but still limited to metastasis larger than 4 mm, in spite of the increased spatial resolution of state-of-the art PET-scanners. Thus, ePLND remains the standard procedure. SLNB increases the detection rate of ePLND and may possess clinical benefits. The positive long-term impact of SLNB requires further investigations to be strongly approved. Whole-body PET/MRI apparently performs similar to PET/CT in the detection of distant metastasis. Hence, the cost-effectiveness in this setting must be further clarified. Moreover, imaging with PET/CT impacts the management in a considerable fraction of patients when performed for primary staging. Future controlled randomized trials are needed to establish a strong recommendation regarding the role of PSMA-targeting PET/CT or MRI in this setting. Finally, radiomics is an encouraging field in the era of high-quality imaging, especially with PET/MRI. More studies are mandatory to determine its accuracy in the evaluation of primary tumor, LN metastasis, and prognosis of PCa.

## Figures and Tables

**Table 1:** Diagnostic performance, advantages, and disadvantages of different radiotracers in the evaluation of the primary staging of prostate cancer.

Radiotracer	Lesion Site	Sensitivity (%)	Specificity (%)	Advantages	Disadvantages	Reference
[^18^F]FDG	T	37–52 67~	- 72~	Providing prognostic information. Incidental uptake is a warning sign. Widely available.	Limitation in well-differentiated PCa. Uptake overlap in malignant and benign lesions. Urinary excretion.	[32,34,35,38,41,44,181]
[^11^C/^18^F]Choline	T	62	76		Non-specific for tumoral lesions. Limited sensitivity. No association between the intensity of uptake with histopathologic or laboratory parameters. Urinary excretion.	[11,54,55,56,57]
	LN	50–59 51 *	92–95 99 *	High specificity. Node-based sensitivity higher than conventional imaging.	Limited value in small LNs.	[56,58,59]
	BM	95	91	Detecting early marrow metastasis. Comparable sensitivity with MRI.	Inferior sensitivity in comparison with Na[^18^F]F PET/CT.	[60,61,62,63]
[^11^C]Acetate	T	93 75 *	- 73 *	Non-urinary excretion. Imaging in 20 min after injection.	Non-specific for tumoral lesions. Limited sensitivity. No association between the intensity of uptake with histopathologic or laboratory parameters. Limited availability.	[12,46,70,71,72,73,74]
	LN	73	-		Suboptimal sensitivity for small LN metastasis.	[12,76]
[^68^Ga]Ga-PSMA	T	70 *	84 *	Moderate sensitivity and high specificity for the detection of local tumor extent. Correlation between SUV_max_ of the primary tumor and GS, PSA and probability of presence of distant metastases.	Limited sensitivity for small lesions. Lower uptake in tumors with lower GS. Urinary excretion.	[81,83,84,85,87,88,89]
	LN	61–84	95–97	High specificity.	Suboptimal sensitivity for small LN metastasis.	[83,92,93,95]
	BM	97	100	Higher sensitivity and specificity compared to bone scan. Superior sensitivity compared to MRI. Comparable diagnostic value compared to Na[^18^F] F PET/CT.		[63,96]
[^18^F]PSMA	T	95–100	-	Lower positron energy/higher spatial resolution. Non-urinary excretion. Good correlation with mpMRI and histopathology.		[107,108,109,111,112,120,121,122]
	LN	87 ^§^ 71.2 *^,§^ 28.1–52.5 ^¤^	98 ^§^ 99.5 *^,§^ 94.0–99.4 ^¤^	High spatial resolution (detection of smaller LNs. Low rate of false positive results.		[108,112,124,126,127,128]
	DM	86–95% ^§^	76–90% ^§^	Superior to bone scan and MRI.	Biliary excretion (limiting detection of liver metastases). Non-specific uptake in bone lesions.	[129,130]
[^18^F]Fluciclovine	T	86.3	75.5		Non-specific for tumoral lesions.	[151,153]
	LN	40	100	High specificity.	Limited sensitivity.	[154,155]
[^99m^Tc]PSMA	T	94–100	-	High accuracy for detecting primary lesions. Maybe helpful for guided biopsy in suspicious patients with negative biopsies.	Urinary excretion.	[17,143,144]
	LN	50	87	Good specificity.	Low sensitivity	[143]
Na[^18^F]F	BM	96	97	High sensitivity	Non-specific agent. Only bone lesions.	[63]
[111In]In- capromab pendetide	LN	62	72		Limited sensitivity. Poor image quality.	[25]
[^18^F]FDHT	T	63	86	Non-invasive evaluation of hormone receptor status. Correlation between a positive scan and higher PSA level.	Low sensitivity. Limited availability.	[161,162]
[^68^Ga]Ga-RM2	T	88	81	High lesion-based sensitivity for primary lesion.		[166]
		70	-		Limited sensitivity for LN metastasis.	[173]

~ in both staging and re-staging; * Lesion-based; ^§^ [^18^F]PSMA; ^¤^ [^18^F]FDCFPyL; AUC: area under curve; BM: bone metastasis; DM: distant metastasis; FDG: fluorodeoxyglucose; FDHT: 16beta-18F-fluoro-5-alpha-dihydrotestosterone; GS: Gleason score; LN: lymph node; mpMRI: multiparametric magnetic resonance imaging; PCa: prostate cancer; PET/CT: positron emission computed tomography/computed tomography; PSA: prostate-specific antigen; PSMA: prostate-specific membrane antigen; SUV_max_: maximum standardized uptake value; T: primary tumor; WD: well-differentiated.
